# Radial motor nerve conduction study in posterior interosseous nerve syndrome and multifocal motor neuropathy

**DOI:** 10.1055/s-0045-1806818

**Published:** 2025-04-27

**Authors:** José Pedro S. Baima, Carlos Otto Heise

**Affiliations:** 1 Universidade de São Paulo, Departamento de Neurologia, São Paulo SP, Brazil.; 2 Universidade Federal do Ceará, Unidade do Sistema Nervoso, Fortaleza CE, Brazil.

**Keywords:** Peripheral Nervous System Diseases, Radial Neuropathy, Nerve Compression Syndromes, Electromyography

## Abstract

**Background** Finger extension weakness could be a presentation of either posterior interosseous nerve (PIN) syndrome or multifocal motor neuropathy (MMN). However, there is a delay in the diagnosis of MMN in cases with a selective radial weakness, as they are frequently misdiagnosed as PIN.

**Objective** To analyze which variables in nerve conduction studies could aid in the early diagnosis of MMN.

**Methods** We reviewed charts of patients with diagnoses of MMN or PIN syndrome, from 2014 to 2022, in a single Brazilian reference center. Electrophysiological parameters included in the analysis were motor conduction velocity (CV), the presence and magnitude of conduction block (CB), distal motor latencies (DML), and the compound muscle action potential amplitude (CMAP) of the affected radial nerve.

**Results** A total of 44 radial nerves were included in the study. Axonal loss was associated with a diagnosis of PIN syndrome, while conduction block was associated with MMN ( *p* < 0.05). No patient with PIN had a CB over 60%, while 7 out of 12 radial CB blocks in patients with MMN were above that. Axonal degeneration was present in 4 MMN patients and in all patients with PIN syndrome. There was no difference in CV and DML between groups.

**Conclusion** The presence of CB or the absence of distal CMAP amplitude reduction should lead physicians to consider MMN, and a comprehensive nerve conduction study should be performed.

## INTRODUCTION

Multifocal motor neuropathy (MMN) is characterized clinically as an asymmetric progressive weakness predominantly in the upper limbs. 1 2 Pure motor weakness must be present for more than one month, but usually longer than 6 months. 3 Weakness in two nerves is required to fulfill core criteria, characterizing a pure motor multiple mononeuropathy. 1 3 In cases where there is only one nerve affected, it should be classified as a possible diagnosis. 3 Extensor muscle weakness of the upper limb is usually more prominent than flexor cases, and this could be a confounding factor, especially with subclinical involvement of other nerves. There are reports in the literature of a 30-year delay in MMN diagnosis due to restricted involvement of the radial nerve. 4 Classically, there is a good response to intravenous immunoglobulins treatment. 5 6 Since it is a treatable condition, and a longstanding disease evolves into axonal loss, early diagnosis and treatment are of paramount importance.

The radial nerve is a motor and sensory nerve located above the elbow and bellow the spiral groove, branching to the brachioradialis muscle and the radial wrist extensors. 7 At the elbow, the radial nerve divides into superficial sensory and deep motor branches called posterior interosseous nerve. 7 The posterior interosseous nerve is responsible for finger and ulnar wrist extension. 8 Entrapment neuropathy of this nerve is a result of compression of the arcade of Frohse in the proximal forearm, but can also be due to inflammatory and traumatic lesions. 8 9 The main symptom of this syndrome is finger extension weakness, and wrist extension is relatively spared. 9

Finger extension weakness is a common presentation of either posterior interosseous nerve (PIN) syndrome or MMN, and electrodiagnostic (EDX) studies are essential in the evaluation of these patients. 10 We have seen MMN individuals diagnosed with posterior interosseous syndrome by hand surgeons. Some of them had already been submitted to surgery with no clinical improvement. A thorough electrodiagnostic evaluation of the four limbs, including the proximal upper limbs segments, would be advisable to avoid this, but would increase time, cost, and discomfort for the patients, and might not be feasible. The objective of this study was to identify possible electrophysiological clues for MMN in patients with selective finger drop.

## METHODS

We reviewed charts and EDX of patients from 2014 to 2022, with a clinical diagnosis of multifocal motor neuropathy (MMN) or PIN syndrome. Diagnosis of PIN syndrome was determined by clinical evolution, and patients could have only unilateral selective involvement of the posterior interosseous nerve, with preserved radial sensory nerve and no proximal radial innervated muscle involvement on electromyography. Patients with MMN were defined by clinical and neurophysiological criteria, similarly to what has been reported in different studies. 11

Patients in the MMN group were included if the radial nerve was affected, but they were allowed to have involvements of other nerves due to small sample size. Exclusion criteria were MMN patients without evidence of radial involvement on EDX, PIN syndrome patients with EDX not consistent with selective involvement, or sensory radial abnormalities. When available, serological antibodies and nerve ultrasounds were reviewed to increase the diagnostic certainty, but they were not available in all patients and therefore were excluded from the analysis.

Electrophysiological parameters included in the analysis were motor conduction velocity (CV), the presence and magnitude of conduction block (CB), distal motor latencies (DML), and the amplitude of the compound muscle action potential (CMAP) of the affected radial nerve recorded over the extensor indicis.

**Figure 1:**
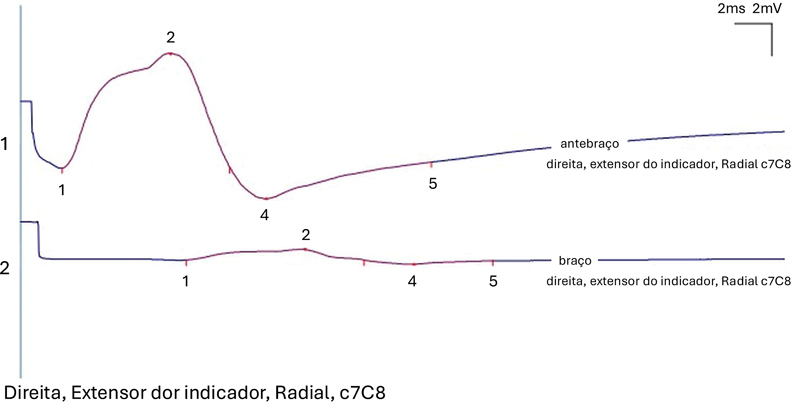
A 63% CB recorded over the extensor indicis in a patient with multifocal motor neuropathy (MMN).

The presence of a conduction block was defined as a CMAP amplitude drop between the forearm and middle arm with a stimulation of more than 30%, if CMAP amplitude was at least 20% above the lower limit, and at least 1 mV. 12 Duration of CMAP ought to be less than 30% to exclude pathological temporal dispersion ( Figure 1 ). Motor axonal degeneration was defined as a distal CMAP amplitude of less than 5 mV in peak-to-peak measurement, according to the normal values of the laboratory. Patients with PIN syndrome were submitted to needle electromyography of the PIN and proximal radial innervated muscles (including triceps brachii and brachioradialis) to confirm their selective involvement by the posterior interosseous nerve. Age at the time of the EDX and gender were included in analysis, due to influence in CMAP amplitudes.

Statistical significance was calculated with the Mann-Whitney U-test for continuous variables. Electrophysiological results were also transformed into normal or abnormal, to be analyzed using the Fisher test for categorical variables. A *p* -value below 0.05 was considered significant.

The institutional review board of Hospital das Clínicas da Faculdade de Medicina da Universidade de São Paulo (HC-FMUSP) approved the investigation protocol, and waived the need for informed consent, since this was a retrospective study, and no patient had undergone an experimental intervention.

## RESULTS

We found 12 patients with MMN, 12 with idiopathic PIN syndrome, and 4 with traumatic PIN syndrome. There were four patients with MMN excluded due to lack of radial nerve involvement; two patients with idiopathic PIN syndrome were excluded due to brachioradialis muscle involvement and rectification of diagnosis as proximal radial neuropathy. Age did not differ between the two groups, with a median of 49 (34–59) years for NMM and 47 (11–82) for PIN syndrome patients ( *p* > 0.05). Gender also did not differ in both samples, with 50% of NMM group and 57% of the PIN syndrome group being male ( *p* > 0.05).

**Figure 2:**
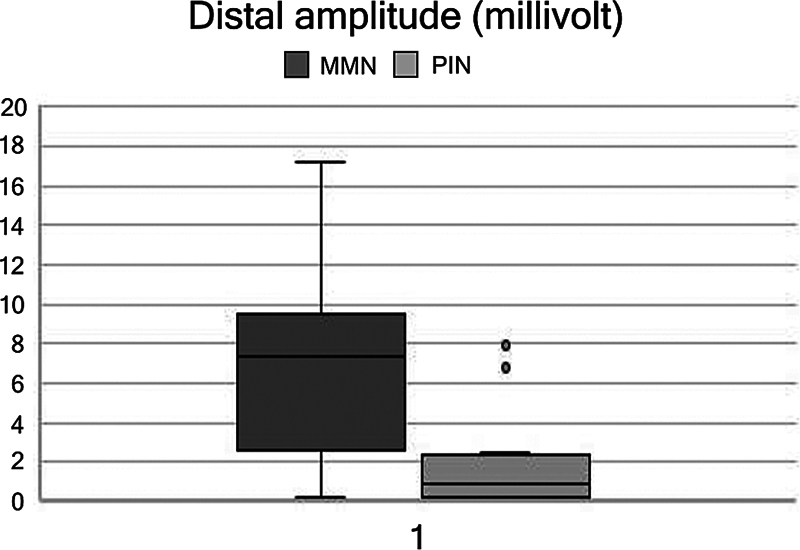
Boxplot of distal amplitude of radial nerve compound muscle action potential in millivolts of MMN (left) and posterior interosseous nerve (PIN) syndrome (right) patients. Axonal loss is greater in PIN syndrome.

**Figure 3:**
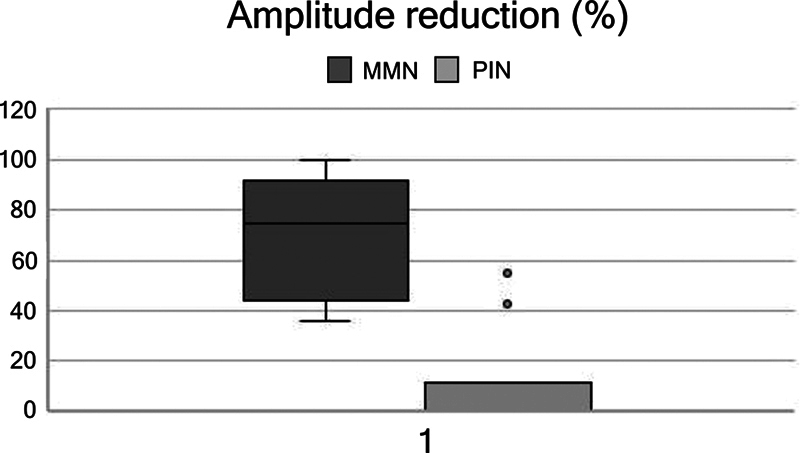
Boxplot of the magnitude of CB in terms of the percentage of patients with MMN (left) and PIN syndrome (right). One can note that CB is significantly higher in MMN.

A total of 44 radial nerves conduction studies were included. The MNN group had 8 patients and the PIN 14. All MMN patients presented with CB in the radial nerve, as well as two of the PIN syndrome patients (Fisher and U Mann-Whitney tests, with *p* < 0.05). Mean CMAP amplitude drop was 76% (36–100) in the MMN group, and 9% (range 0–43) in the PIN syndrome group. No patient with PIN syndrome had a CB over 60%, while 7 out of 12 radial nerve conduction blocks in patients with MMN were above that percentage ( Figure 2 ). Axonal degeneration was present in 4 patients with MMN and in all patients with PIN syndrome (Fisher and U Mann-Whitney with a *p* < 0.05) ( Figure 3 ). The median distal CMAP amplitude in the MMN group was of 6.6 mV (range: 0.2–17.2), and median CMAP in the PIN syndrome group was of 1.8 mV (range: 0–7.9). Differences in DML or motor CV of the radial nerve were not statistically significant ( Table 1 ).

**Table 1:** Statistical analysis of electrophysiological parameters

Parameter	Fisher	Mann-Whitney U	*p* < 0.05
CV	0.4003	0.09	No
CB	0.0001	<0.00001	Yes
Distal CMAP	0.0142	0.00736	Yes
DML	0.0953	0.101	No

Abbreviations: CV, conduction velocity; CB, conduction block; CMAP, compound muscle action potential; DML, distal motor latency.

## DISCUSSION

Our aim was to test the clinical impression that patients with axonal degeneration on motor nerve conduction studies probably had an isolated posterior interosseous syndrome, while patients presenting with conduction block were more likely to have multifocal motor neuropathy. Our findings are consistent with this observation, statistically significant, and have the potential to guide exam protocols on everyday practice.

Because of the frequency, focal neuropathies are one of the most important differential diagnoses of MMN. 13 As previously mentioned, there are reports in the literature of a 30-year delay in the diagnosis due to restricted involvement of the radial nerve. 4 In this series, one of the cases had a 20-year diagnostic delay, and the patient has been submitted to two surgeries without clinical benefit. Overall, clinical hypothesis of MMN is also little considered. For instance, in a series of 46 patients, only 6 were initially referred with this hypothesis. 14 A clinical clue to differentiate MMN from PIN syndrome is the finger extensor muscle weakness within the same muscle. 15 This sign reflects that fingers innervated by the same posterior interosseous nerve have different grades of weakness, and a possible explanation is fascicular involvement in MMN. 15 Data on this finding could not be retrieved from the charts, but it should be taken into consideration in future assessments of patients during nerve conduction.

The CB can be measured by the ratio of the amplitude drop or by the drop of CMAP area. The latter is the classical definition, as stated by the criteria, but the first has more sensitivity in real world data. 3 11 We chose to use the proximal to distal ratio of amplitude, as defined by the American Academy of Neuromuscular and Electrodiagnostic Medicine. 16 17

Intuitively, CB is associated with weakness, but approximately one-third of abnormalities in a series of cases were found in non-weakened muscles. 18 This could lead to complications, because a limited protocol of nerve EDX studies could miss conduction blocks in unsuspected nerves. The finding of conduction block outside typical entrapment sites should drive to the hypothesis of MMN. 13 Although most studies describe an axonal pattern in nerve conduction studies as typical for PIN syndrome, there are also reports indicating that CB can be a finding in this syndrome. 19 20

**Figure 4:**
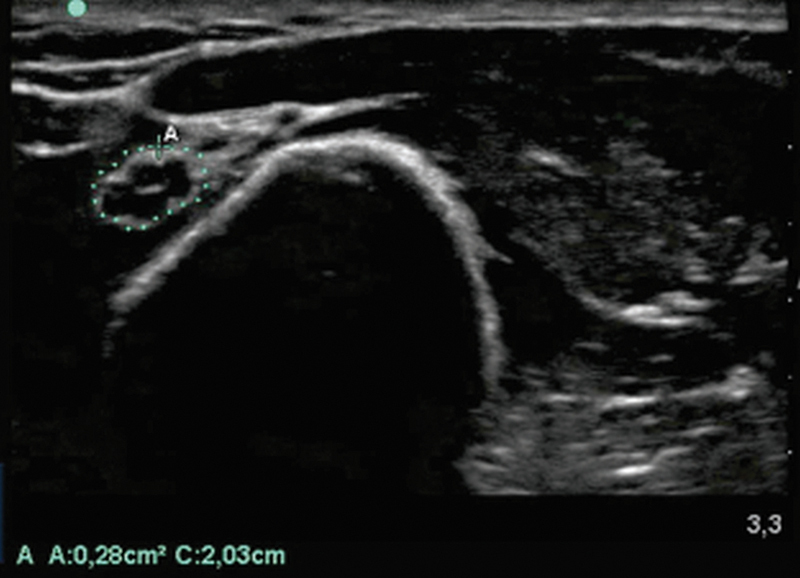
Note: Reference value – < 10mm ^2^ . Mid-arm radial nerve enlargement in a patient referred to neurophysiologic evaluation with PIN syndrome diagnosis, who actually had MMN.

Nerve ultrasound is also a tool for assessment that can identify nerve enlargement in NMM, posterior interosseous nerve enlargement at compression site, or even other sources of compression. 21 An ultrasound performed during nerve conduction studies in one of the patients with finger extension weakness demonstrating enlargement compatible with MMN is depicted in Figure 4 . A previous study did not find correlation between ultrasonographical abnormalities and clinical weakness. 22 A recent publication has demonstrated that nerve ultrasound was able to identify abnormalities in unsuspected nerves in patients with motor mononeuropathies and were able to suggest a more widespread immune etiology. 23 Similarly to these findings, we propose in this work that, through the identification of CB, nerve conduction studies could suggest a more widespread immune process and increase suspicion of MMN. Although ultrasound has a proven value in mononeuropathy and could be useful in diagnosis of atypical cases of MMN, it is not widely available, and not all neurophysiologists are trained in ultrasound. 24 Anti-GM1 igM antibodies are very helpful to confirm the diagnosis of MMN, but a nationwide case series in Japan only found their presence in 54% of the cases. 25

Although anti-GM1 antibodies and nerve ultrasound are useful in the differential diagnosis, negative antibodies assay or absence of nerve focal enlargement outside the posterior interosseous nerve cannot exclude the diagnosis in all cases and are not easily available in resource-limited areas. In contrast, nerve conduction study is an established method available throughout the globe. Additionally, it is often one of the first methods to investigate peripheral neuropathy.

The limitations of this study are the small number of cases and the fact that its retrospective nature. It is possible that some patients with idiopathic PIN syndrome diagnosis could eventually develop MMN. Due to our limited sample, we could not restrict patients with motor MMN presenting only with radial weakness. These are both rare conditions, so prospective controlled studies are difficult to perform.

In conclusion, MMN is a rare disease, and its diagnosis is often missed in mild cases. Patients clinically presenting selective radial involvement are at risk of misdiagnosis of posterior interosseous syndrome. When evaluating patients with suspected PIN syndrome, have a CB over 60% and the absence of distal CMAP amplitude reduction, MMN should be considered. We also recommend testing other motor nerves, including proximal segments even in non-weakened muscles, and looking closely for conduction block or other motor nerve abnormalities.
